# Corrigendum to “UBE2C Induces Cisplatin Resistance via ZEB1/2-Dependent Upregulation of ABCG2 and ERCC1 in NSCLC Cells”

**DOI:** 10.1155/2020/5025641

**Published:** 2020-10-20

**Authors:** Yan Wu, Dan Jin, Xiaohong Wang, Jing Du, Weihua Di, Jiajia An, Cuijie Shao, Jiwei Guo

**Affiliations:** ^1^Cancer Research Institute, Binzhou Medical University Hospital, Binzhou 256603, China; ^2^Department of Pain Medicine, Binzhou Medical University Hospital, Binzhou 256603, China; ^3^Department of Thyroid and Breast Surgery, Binzhou Medical University Hospital, Binzhou 256603, China; ^4^Department of Clinical Laboratory, Binzhou Medical University Hospital, Binzhou 256603, China

In the article titled “UBE2C Induces Cisplatin Resistance via ZEB1/2-Dependent Upregulation of ABCG2 and ERCC1 in NSCLC Cells” [1], there is a concern of figure issue. In Figure 1(c), the wound healing images of PBS treatment were unintentionally misused for the control, siUBE2C, siUBE2C + ZEB1, and UBE2C treatments in the groups of 0 h. In addition, the wound healing image of siUBE2C treatment was unintentionally misused for the UBE2C + siZEB1 treatment in the groups of 36 h. The only change is in the panel of Figure 1(c), and the rest of the figure is identical to the published version. This unintentional error also has no bearing on the work's scientific conclusions in any way. The authors apologize to the Editor of *Journal of Oncology* and to the readership for any inconvenience caused. The corrected figure and legend are presented here.

## Figures and Tables

**Figure 1:**
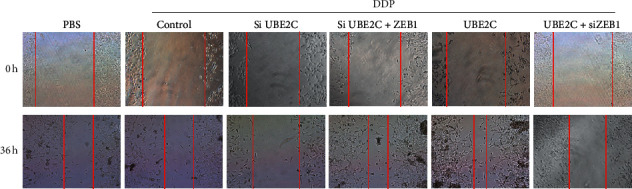
Knockdown of ZEB1/2 inhibits UBE2C-dependent cellular growth, invasiveness, and EMT in DDP-resistant NSCLC cells. (c) Scratch assay indicated that UBE2C promotes cell migration via regulating ZEB1 in A549/DDP cells with treatment of DDP at 6 *μ*g/ml for 36 h. Results were presented as mean ± SD, and the error bars represent the SD of three independent experiments. ^*∗*^*p* < 0.05; ^*∗∗*^*p* < 0.01 versus control group.
